# Hypopituitarism manifesting after invasive dental treatment in a patient with carcinoma of the tongue: a case report

**DOI:** 10.1186/s12903-020-01082-x

**Published:** 2020-04-15

**Authors:** Yu Ohashi, Naoko Tsunoda, Kei Onodera, Shin Iijima, Ikuya Miyamoto, Toshimi Chiba, Hiroyuki Yamada

**Affiliations:** 1 Division of Oral and Maxillofacial Surgery, Department of Reconstructive Oral and Maxillofacial Surgery, Iwate Medical University School of Dentistry, 19-1, Uchimaru, Morioka, Iwate 020- 8505 Japan; 2 Division of Internal Medicine of Dentistry, Department of Oral medicine, Iwate Medical University School of Dentistry, 19-1, Uchimaru, Morioka, 020-8505 Japan

**Keywords:** Tongue cancer, Hypopituitarism, Dental treatment, Case report

## Abstract

**Background:**

The symptoms of hypopituitarism are not usually discussed in the clinical setting of oral surgery.

**Case presentation:**

We herein report a case of hypopituitarism that became evident after biopsy and extraction of several teeth in a 68-year-old man with tongue cancer. Three days after biopsy, the patient developed nausea and vomiting, and his serum sodium had fallen to 124 mEq/L. His serum cortisol concentration was low. Although the plasma concentration of adrenocorticotropic hormone (ACTH) was within the normal range, ACTH stimulation testing showed a lack of cortisol response. Given these findings, we suspected secondary adrenal insufficiency. To investigate the cause of secondary adrenal insufficiency, MRI of the head was performed, which revealed pituitary gland atrophy. The results of pituitary anterior lobe hormone-stimulation tests were compatible with hypopituitarism. Thirty days after biopsy, partial tongue resection was successfully performed under general anesthesia with perioperative hydrocortisone supplementation.

**Conclusions:**

We must be aware of various signs of hypopituitarism when we perform invasive dental treatment.

## Background

Hypopituitarism is a deficiency in one or more trophic hormones, including adrenocorticotropic hormone (ACTH), thyroid-stimulating hormone (TSH), prolactin, growth hormone (GH), and gonadotropins, such as luteinizing hormone (LH) and follicle-stimulating hormone (FSH) [1]. The clinical symptoms depend on the hormone affected and its level of deficiency. Traumatic brain injury and subarachnoid hemorrhage are well-established causes of hypopituitarism. However, this risk is not fully recognized by clinicians [2].

The most common type of hypopituitarism is LH and FSH deficiency, which results in amenorrhea in women and decreased libido in men [3]. However, these symptoms are not usually discussed in the clinical setting of oral surgery. Moreover, routine preoperative screening tests generally include no testing of endocrine hormones if patients do not report subjective symptoms. Therefore, untreated hypopituitarism without marked symptoms is easily overlooked.

We experienced a case of hypopituitarism that manifested after invasive dental treatment, a clinical situation rarely documented in the literature. We herein report a case of hypopituitarism that became evident after biopsy and extraction of several teeth in a patient with tongue cancer.

## Case presentation

**Fig. 1:**
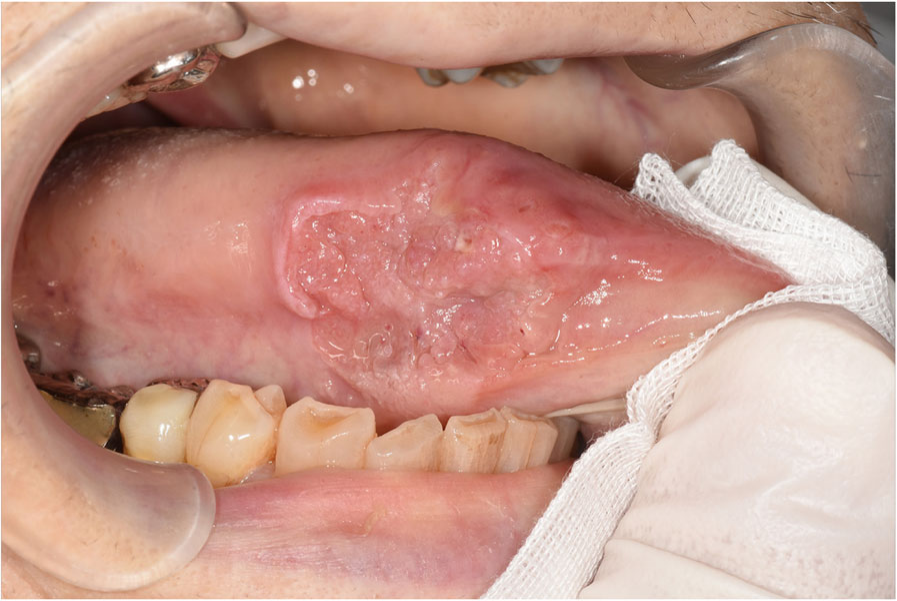
Photograph showing the lesion on the right edge of the tongue

A 68-year-old man was referred to the Department of Reconstructive Oral and Maxillofacial Surgery at Iwate Medical University School of Dentistry on May 15, 2017, for diagnosis and treatment of an ulcer on the right side of the tongue. He had first noticed the ulcer about 1 month earlier. The patient’s medical history included loss of consciousness resulting from dysautonomia at the age of 32. There was no history of traumatic injury or radiotherapy. Family history was unremarkable. Physical examination revealed facial pallor. In the oral cavity, an ulcer measuring 28 × 18 mm was present on the right edge of the tongue (Fig. 1). The ulcer was indurated on palpation. There was no cervical lymphadenopathy. The results of blood testing are shown in Table 1. Serum electrolytes were within the normal range.

**Table 1:**
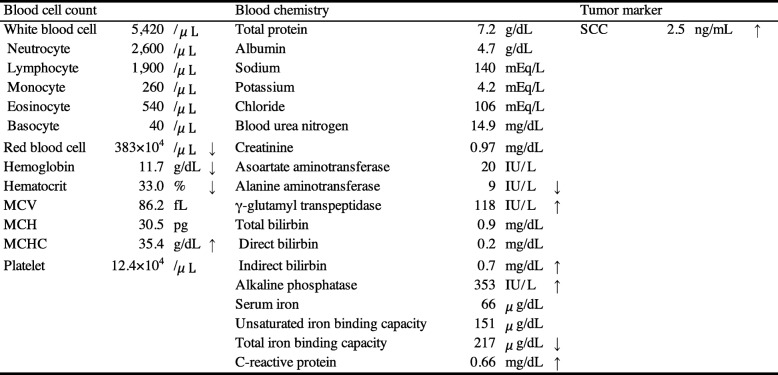
Blood examination on first admission

*MCV* mean corpuscular volume, *MCH* mean corpuscular hemoglobin, *MCHC* mean corpuscular hemoglobin concentration, *SCC* squamous cell carcinoma, *fL* femto litre, *pg* pico gram, *IU* international unit, *ng* nano gram

Magnetic resonance imaging (MRI) was performed with a 3.0-Tesla system (MR750; General Electric Company, Boston, MA, USA). On T1-weighted axial images, the mass on the right edge of the tongue was isointense relative to muscle. On T2-weighted images, slightly increased signal intensity was noted within the mass. On gadolinium-enhanced T1-weighted images, the mass was homogeneous and highly enhanced.

Positron emission tomography was performed with a Discovery PET/CT 600 scanner (General Electric Company, Boston, MA, USA). The image showed abnormal accumulation of fluorodeoxyglucose on the right edge of the tongue at the location of the mass. There was no abnormal accumulation of fluorodeoxyglucose in the lymph nodes or any other organ.

**Fig. 2:**
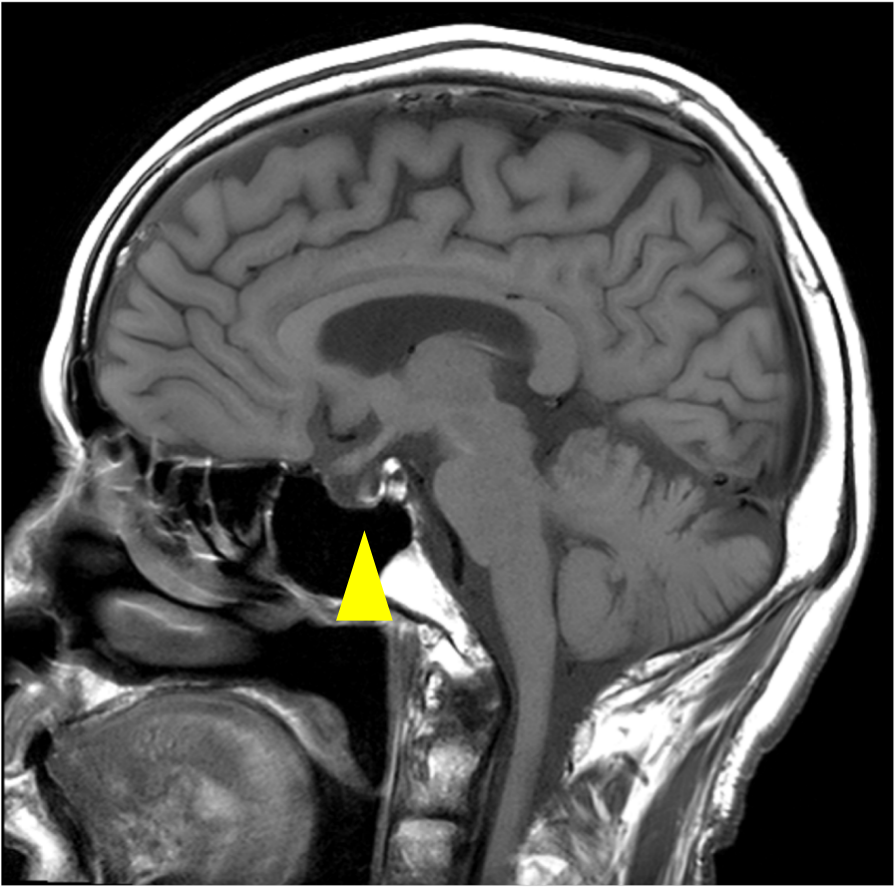
Brain MRI findings. T2-weighted sagittal image showing atrophic pituitary gland (arrowhead)

**Fig. 3:**
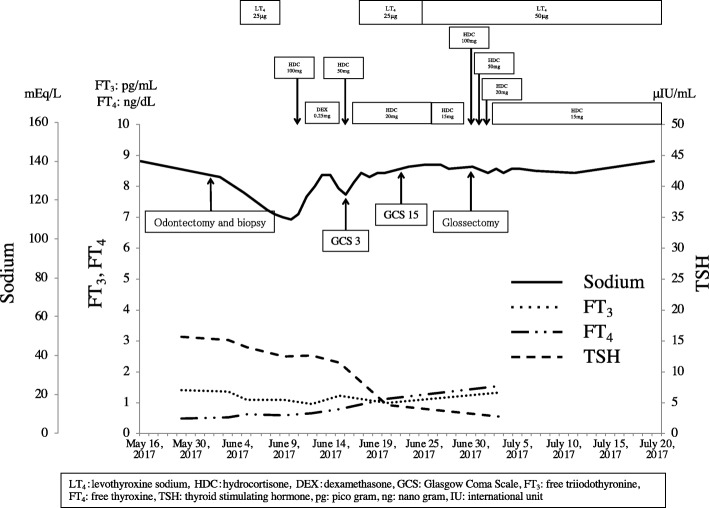
Clinical course

The patient was hospitalized on May 26. On the basis of a clinical diagnosis of tongue cancer (cT2N0M0), biopsy was performed under local anesthesia. During the same procedure, several teeth that were mechanically stimulating the lesion were extracted. The serum sodium concentration was 132 mEq/L on the day of biopsy. Three days after biopsy, the patient developed nausea and vomiting, and his serum sodium had fallen to 124 mEq/L. In addition, laboratory examinations (Table 2) showed high serum TSH, low free triiodothyronine (FT_3_), and low free thyroxine (FT_4_). Because antithyroid peroxidase antibodies and antithyroglobulin antibodies were confirmed as positive, a diagnosis of Hashimoto thyroiditis was made. Administration of levothyroxine sodium (LT_4_) was started. However, nausea and vomiting were not controlled. The findings of low serum cortisol, low serum sodium, and high urine osmolality raised suspicion of acute adrenal insufficiency. The patient was transferred to the medical department and administration of dexamethasone at 0.25 mg per day was started instead of LT_4_. The plasma ACTH concentration (8.8 pg/mL) was within the normal range. On June 7, ACTH stimulation testing was performed. The plasma cortisol concentration before the test was 1.8 μg/dL. Plasma cortisol concentrations 30 and 60 min after administration of corticotropin (250 μg) were 4.0 μg/dL and 4.8 μg/dL, respectively. On the basis of these findings, we suspected secondary adrenal insufficiency. On June 13, administration of LT_4_ was restarted for the treatment of hypothyroidism. To investigate the cause of secondary adrenal insufficiency, MRI of the head was performed, which revealed pituitary gland atrophy (Fig. 2). The results of pituitary anterior lobe hormone-stimulation tests are listed in Table 3. These results were compatible with hypopituitarism. The patient’s severe consciousness disorder, which scored 3 on the Glasgow Coma Scale 14 days after biopsy, gradually improved, with full recovery on day 20 after biopsy. Thirty days after biopsy, partial tongue resection was successfully performed under general anesthesia with perioperative hydrocortisone supplementation. The histopathological diagnosis was squamous cell carcinoma of the tongue. The postoperative course was uneventful. The perioperative clinical course is summarized in Fig. 3.

**Table 2:**
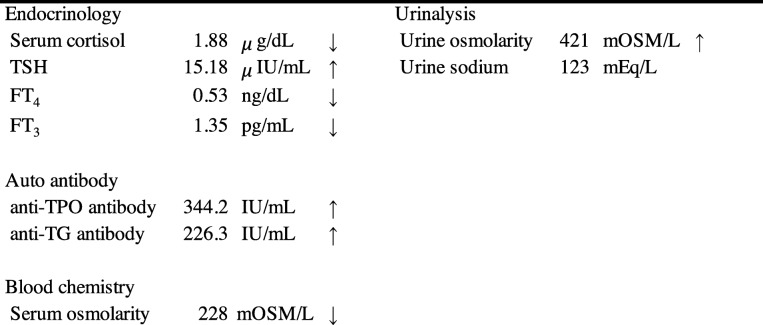
Laboratory findings

*TSH* thyroid stimulating hormone, *FT4* free thyroxine, *FT3* free triiodothyronine, *TPO* thyroid peroxidase, *TG* thyroglobulin, *IU* international unit, *ng* nano gram, *pg* pico gram, *OSM* osmole

**Table 3:** Anterior pituitary function test

	normal value	pre loading	15 min	30 min	45 min	60 min	90 min	normal reaction	decision
ACTH (pg/mL)	7〜56	9.1	–	15.1	–	21.2	20.8	more than diploid number (30–60 min)	hyporesponsiveness susp
Cortisol (*μ*g/dL)	4〜23.3	4.4	–	4.8	–	5.1	5.1	more than 18 (60 min)	hyporesponsiveness
TSH (μIU/mL)	0.35〜4.94	1.29	–	2.98	–	2.77	2.51	3.5–15 (30 min)	hyporesponsiveness
PRL (ng/mL)	1.4〜14.6	2.7	–	4.4	–	4.1	3.7	more than diploid number (15–30 min)	hyporesponsiveness
LH (mIU/mL)	0.7〜24.2	0.6	–	1.5	–	2.1	2.1	five times the value (30 min)	hyporesponsiveness
FSH (mIU/mL)	2.7〜56.7	1.8	–	2.0	–	2.3	2.4	1.5–2.5 times the value (30 min)	hyporesponsiveness
GH (ng/mL)	0.68〜8.7	0.40	1.17	0.98	0.70	0.49	–	more than 9 (15–30 min)	hyporesponsiveness

*ACTH* adrenocrticotropic hormenoe, *TSH* thyroid-stimulating hormone, *PRL* prolactin, *LH* luteinizing hormone, *FSH* follicle-stimulating hormone, *GH* growth hormone, *pg* pico gram, *IU* international unit, ng: nano gram

## Discussion and conclusions

Most cases of hypopituitarism arise from destructive processes directly involving the anterior pituitary, including tumors, traumatic brain injury, Sheehan syndrome, apoplexy, inflammatory disorders, and radiation [1]. Brain injury resulting from traumatic thoracic injury [4], autoimmune disease [5], and metastasis to the pituitary [6] are additional reported causes of hypopituitarism. Recently, immune checkpoint inhibitors have been used to treat various types of cancer. With increased use of these inhibitors, physicians should be aware of the possibility of immune checkpoint inhibitor-induced hypophysitis. Cytotoxic T-lymphocyte antigen (CTLA)-4 is expressed in the pituitary gland; anti-CTLA-4 antibodies were reported to induce hypophysitis [7]. Furthermore, anti-programmed cell death (PD)-1 and anti-PD-ligand 1 antibodies have been reported to induce hypophysitis, leading to pituitary atrophy [8]. Therefore, we must be aware of various signs of hypopituitarism when we perform invasive dental treatment.

The anterior lobe of the pituitary has high functional reserve; therefore, more than 75% of the parenchyma must be lost before symptoms of hypopituitarism are seen [1]. In the present case, anterior lobe hormone-stimulation tests revealed hyporeactivity of ACTH, TSH, LH, FSH, prolactin and GH. MRI revealed pituitary atrophy. These results and the medical history of loss of consciousness indicate that the patient’s hypopituitarism might have been gradually progressive before dental treatment. The invasive dental treatment might have decreased the functional reserve of the pituitary, resulting in clinical symptoms.

It is rare that an invasive medical procedure results in diagnosis of hypopituitarism. To the best of our knowledge, two such cases have been reported in the Japanese literature [9, 10]. In these cases, food intake decreased as a result of invasive medical procedures, and the stress of hospitalization increased the relative cortisol requirement. These factors caused adrenal insufficiency to become evident. Hypopituitarism with resulting adrenal insufficiency causes malaise, fatigue, nausea, vomiting, weight loss, and muscle weakness. Adrenal crisis is biochemically characterized by hyponatremia and hypoglycemia in patients with hypopituitarism. Hyperkalemia is not present in hypopituitary patients because they do not have mineralocorticoid deficiency, unlike patients with primary adrenal insufficiency [11]. Surgical stress can result in an adrenal crisis, with shock or disturbed consciousness as symptoms. These symptoms are life-threatening; therefore, urgent administration of hydrocortisone is required [12]. In the present case, the patient’s food intake decreased after invasive dental treatment, resulting in worsening hyponatremia. We attributed the patient’s symptoms to severe pain after dental treatment; however, these symptoms were caused by hypopituitarism with adrenal insufficiency.

Treatment of hypopituitarism is classified as causal or symptomatic. In the present case, neither pituitary surgery nor radiation had been performed, and the cause of hypopituitarism was not obvious; therefore, symptomatic treatment was selected. Because hypopituitarism was diagnosed before the treatment of tongue cancer, adequate perioperative hydrocortisone supplementation was administered. As a result, adrenal crisis was avoided during surgery for tongue cancer.

In conclusion, when decreased appetite, malaise, and fatigue occur after invasive treatments, the possibility of masked hypopituitarism should be considered.

## Data Availability

Not applicable.

## Abbreviations

MRI: Magnetic resonance imaging
ACTH: Adrenocorticotropic hormone
PET: Positron emission tomography
CT: Computed tomography
TSH: Thyroid-stimulating hormone
TPO: Thyroid peroxidase
TG: Thyroglobulin
FT_3_: Free triiodothyronine
FT_4_: Free thyroxine
PRL: Prolactin
LH: Luteinizing hormone
FSH: Follicle-stimulating hormone
GH: Growth hormone
LT_4_: Levothyroxine sodium
HDC: Hydrocortisone
DEX: Dexamethasone
GCS: Glasgow coma scale
CTLA: Cytotoxic T-lymphocyte antigen
PD: Programmed cell death
MCV: Mean corpuscular volume
MCH: Mean corpuscular hemoglobin
MCHC: Mean corpuscular hemoglobin concentration
SCC: Squamous cell carcinoma
fL: femto litre
pg: pico gram
IU: International unit
ng: nano gram
OSM: Osmole
